# Strategies to prevent medical errors by nursing interns: a qualitative content analysis

**DOI:** 10.1186/s12912-024-01726-1

**Published:** 2024-01-17

**Authors:** Nastaran Heydarikhayat, Nezar Ghanbarzehi, Kimiya Sabagh

**Affiliations:** 1 Nursing Department, School of Medicine, Iranshahr University of Medical Sciences, Iranshahr, Iran; 2 Department of Pediatric Nursing, School of Nursing and Midwifery, Zahedan University of Medical Sciences, Zahedan, Iran

**Keywords:** Medical errors, Qualitative study, Student, Nursing, Internship

## Abstract

**Background:**

Nursing interns often face the serious challenges and stress of clinical training. Identifying effective strategies in reducing medical errors can improve student performance and decrease patient risk and injury from errors. The purpose of this study was to identify strategies to prevent medical errors by nursing interns in Medical Universities in Sistan and Baluchistan, Southeast of Iran.

**Methods:**

This is a qualitative study using a content analysis approach. Purposive sampling was used. The study was conducted in 3 medical universities. Ten nursing interns participated in this study. Open-ended, semi-structured, and face-to-face, interviews were used to explore the experience of nursing interns about strategies to prevent medical errors during their internship.

**Results:**

Findings include 20 subcategories, 6 categories and one theme. The main theme is “strategies to prevent medical errors during internship”. Six categories included “strategies to prevent medical errors during internship”. These included “Professional acceptance and support”, “Revision of the implementation of the educational curriculum”, “Retraining courses for challenging skills”, ”Creating learning opportunities” “Professionalization”, and “Facilities and requirements”.

**Conclusions:**

Preventing medical errors requires different strategies before and during nursing internship. Error prevention strategies include retraining and preparatory courses for challenging areas, evaluation of students’ performance, and accepting students as members of the health care team, respecting and supporting them and protecting their rights. Learning from medical errors, analysis and reflection on errors should be part of the curriculum during the internship.

**Supplementary Information:**

The online version contains supplementary material available at 10.1186/s12912-024-01726-1.

## Background

Nursing is recognized as an important practice-oriented profession for improving the health of society. Society needs knowledgeable, qualified and competent personnel. In this respect, nursing education also has a mission to develop competent human resources to meet the needs of society. Based on this, the primary task of nursing schools is to train competent and qualified nurses [1]. The educational program in this field consists of two parts including theoretical and practical training. Clinical training provides students with the opportunity to apply theoretical knowledge to a real-world settings and gain more experience and skills [2]. As a link between education and the clinical environment, internship is an important part of nursing education. It is an opportunity for nursing students to apply what they have learned in the clinical settings [3, 4]. Internships help students establish a professional identity and gain independence, self-efficacy, and self-confidence. Weaknesses, inadequacies, and deficiencies in the field of clinical education affect the quality and quantity of medical services and, in turn, the health of society [2]. An important and well-known indicator of the quality of medical and nursing services is patient care and safety standards [5]. Patient safety has been highlighted as a major concern in the healthcare system, with errors not only being the greatest threat to patient safety, but also being considered an important indicator of the quality of care. Medical team members can make errors when providing patient care. These errors occur in a variety of health care professions, but nurses are more likely to make them than other health care professionals [6, 7]. These errors can occur at any stage of the patient-related process and can result in patient injury or death. Therefore, reducing unwanted and inevitable errors is critical to building a secure system of delivering medical services and improving the quality of care in health centers [8].

Medical errors occur when a healthcare provider neglects to provide appropriate treatment or omits to take necessary actions, resulting in harm, injury, or death to a patient [5, 9]. The occurrence of errors leads to negative outcomes such as increased patient mortality, longer hospital stays, increased costs, dissatisfaction and reduced trust in the system [10]. Clinical errors occur in 1 in 10 people [11]. Each year, 400,000 hospitalized patients are injured and 100,000 die from medical errors. Additionally, medical errors cost $20 billion annually [12].

Unlike experienced nurses, interns in developing countries often face the perilous challenges and stress of clinical training [13]. Being in a clinical setting as an experiential learning environment can be a challenging experience for undergraduate [14]. Errors made by nursing students are often caused by factors such as lack of knowledge and high professor-to-student ratios, and limited experience and skills [15, 16]. Medical error rates among students appear to be higher than reported. Despite being important, half of students who commit medical errors don’t report them [17, 18]. The main reasons students gave for not reporting errors were fear of the consequences of reporting, fear of impact on the grade or score, and lack of positive feedback after reporting [19]. A culture of punishment is not effective for medical errors, so solutions should be sought to detect and prevent the occurrence of errors [12]. Consequently, teaching methods should be changed, students should be monitored, non-punitive strategies should be used to reduce the incidence of errors, and reporting should be timely [20].

Many studies have paid attention to the role of error control in preventing and reducing errors, but the main step in this regard is to identify the factors that affect the effective control of medical errors [21, 22]. Recognizing adverse events, learning from them, and working to prevent them can improve patient safety [12]. Since clinical internship is a necessary step for nursing students to graduate and become a qualified nurse [21], and as the possibility of error always exists, special attention must be paid to patients’ health [23].

Identifying effective strategies to reduce errors can improve student performance and reduce the risk and patient injury caused by errors. Several studies have been conducted on medication errors, medication administration, and even the prevention of medication errors by nurses or students [24–27], but limited research has been conducted on preventive strategies of medical errors by nursing interns who have different working conditions. One study shows that although quantitative methods are still needed, they are generally insufficient to answer complex research questions about health systems [28]. Because the purpose of qualitative research is to understand phenomena and ask why and how questions [29], this method can better articulate the concept of error prevention from experiences of nursing students during internships. Therefore, the purpose of this qualitative study was to identify strategies to prevent medical errors made by nursing interns at medical universities in Sistan and Baluchistan, Southeast of Iran.

## Methods

### Study design and setting

This is a qualitative study using a conventional content analysis approach. Content analysis is a research method that allows data to be analyzed at both the manifest and latent levels. It considers meaning and makes inferences from data [30, 31]. This study was conducted from July 2021 to February 2022. The purpose of the study was to explore the strategies to prevent medical errors among nursing interns. The study setting was southeastern medical universities, such as Zahedan, Zabol and Iranshahr medical universities, which met the inclusion criteria.

### Sampling and participants

Participants were 4th year nursing students who met the following inclusion criteria: Being a nursing student, in their 7th or 8th nursing semester, married or single, in both genders, with 3 educational levels: weak, average, good, based on the cumulative average score of courses passed in all semesters. Purposive sampling was used. Students who met the inclusion criteria were invited to participate in this study. For this purpose, a form was developed that included demographic and educational information as well as experience with and without a challenging and problematic internship period. Each form contained a code created based on the intern’s list. In addition, nursing interns were contacted by phone based on their problematic or non-problematic internship period. The study included students of both sexes, marital status, and from all three educational levels who reported a challenging internship period in the seventh and eighth semesters. Students who did not describe the internship as challenging also participated in the study in order to obtain detailed information about it. A total of 10 nursing interns participated in this study, with a total of 11 interviews conducted. One participant requested a second to provide more information. To consider maximum transferability, nursing interns were selected from first to third-tier universities. Sampling continued until data saturation, meaning that repeated data were collected without extracting new codes. In qualitative research, saturation is an indication of the end of data collection or analysis, the absence of additional data or new codes, the absence of development of new categories, and the extraction of similar instances ensure data saturation [32]. In the present study, data saturation was achieved at interview number 8, but interviews were continued until interview number 11 for greater certainty.

### Data collection

After obtaining the necessary permits, students’ lists were drawn up and phone calls were made. The purpose and methods of the study were explained to participants. Qualified students interested in participating in the study were invited to schedule an interview time and location. Interviews were conducted both in hospital departments and in university settings according to participants’ preferences. A sample interview has been provided as a supplementary file. Open-ended, semi-structured, face-to-face interviews were used to elicit medical errors preventing strategies during nursing internship at medical teaching hospitals. The main questions in the interview guide are: “Have you ever experienced any medical errors during your internship”, “Can you describe the working conditions of intern nurses on the wards?”, “Can you explain the circumstances that led to these medical errors” and “What strategies should be adopted to prevent medical errors made by nursing interns”?. Additionally, probing questions were asked, such as “Can you explain further?” and “Can you give me an example?”. All interviews were conducted by the first and third authors. Interviews lasted from 40 to 60 min, in one or two sessions depending on the participant’s request. All interviews were audio recorded after obtaining permission from all participants in this study. Each interview was listened to several times and then, as quickly as possible, transcribed verbatim.

### Data analysis

MAXQDA software version 20 was used to regularly compare different data and facilitate data organization. Along with data collection, analysis is performed using conventional content analysis method and following the steps suggested by Graneheim and Lundman. The main parts of qualitative content analysis are the unit of analysis, the meaning unit, and abstraction, which includes the creation of codes, categories, and themes [33]. Data analysis was performed following the steps suggested by Graneheim and Lundman. First, the interview is written down as soon as possible after it’s over; next, the interview transcript is read several times to get the general idea; then, units and semantic codes are identified; similar codes are grouped into comprehensive categories and hidden content in the data is extracted [34]. In this method, data analysis begins by reading the text multiple times to achieve a general understanding. In the next step, the entire interview was transcribed verbatim into a Microsoft Word document. Each transcribed interview is considered a unit of analysis. Each text was checked by the same interviewee and revised as necessary. An inductive coding format was used. Initial codes are then determined by identifying the important phrases and underlining them. Meaning units were extracted, classified, and summarized based on similarities and differences. In the next step, the meaning codes were extracted. Then, similarities and differences in the codes are examined to classify them into themes, categories, and subcategories. The final results, including the extracted codes and categories were reviewed by all members of research team [35, 36].

### Trustworthiness

The Lincoln and Guba criteria [37] were used to evaluate the trustworthiness and quality of the findings using several procedures. Long-term involvement in the research process and sample diversity were achieved to ensure credibility. For this purpose, data were collected from nursing students in both the 7th and 8th semesters, based on their gender, marital status, and student’s academic status. Data transferability was achieved through detailed descriptions of the context, participants, and data collection and analysis process. Detailed descriptions of the data collection phases and audit trail were included for greater dependability. Three doctoral nurses out of the study independently reviewed the extracted codes, subcategories, and categories. Confirmability was ensured by member check, i.e., participants read and checked the extracted codes and categories. All participants reviewed the final structures of statements and some adjustments were suggested and made within the categories.

### Quantitative results

Ten interns with an average age of 23.10 ± 1.44 years participated in this study. 70% of the participants were married and none had children. Additional demographic information is presented in Table 1.

**Table 1:** Characteristics of participants in the study (*n* = 10)

Participants number	Age (year)	Sex	Educational semester	Marital status	Part time job
1	24	Male	8	Single	No
2	23	Male	8	Single	No
3	26	Male	7	Married	Yes
4	22	Male	8	Married	No
5	23	Female	7	Married	No
6	22	Female	8	Married	No
7	24	Female	7	Married	No
8	22	Female	8	Married	No
9	21	Female	7	Single	No
10	24	Female	8	Married	No

### Qualitative results

Interview times ranged from 35 to 55 min (average: 42.30 ± 7.55 min). First, 630 primary codes were extracted. Integrating similar codes resulted in 470 codes, 20 subcategories, 4 categories and 1 theme, Table 2. Details of these results are shown in Table 3.

**Table 2:** Extracted subcategories, categories, and theme based on the strategies to prevent medical errors during internship

Category	Subcategories	Examples of codes	Examples of meaning units	Examples of quotations
Professional acceptance and support	Accepting the rights and job descriptions of the interns	- Unreasonable expectations - Disproportionate workload / difficult internship situations -Lack of drug information - fatigue during shift	- Being shocked by the nurse’s high expectations -Lack of fit between workload and students’ abilities - Not knowing all the drugs prescribed for the patient - Fatigue when giving medications to patients	*“I was shocked that a nurse with two or three patients expected me, a student who didn’t know all the medicines, to give all the medicines to the patients. Many times we were too tired to give the medicines patients”.*
	Supportive atmosphere	-Helplessness -Staying alone -Support less Stressful workplace	-Lack of nurses’ help -Nurses’ rejection to help students -Feeling alone in the workplace -Pain and worry due to lack of support	*“Nurses don’t help us, saying, you have to be independent or we don’t have time. That feeling of being alone and without support was really painful and worrying”.*

**Table 3:** Participants’ experiences of strategies to prevent medical errors by nursing interns

Theme	Categories	Sub-categories
Strategies to prevent medical errors during internship	Professional acceptance and support	Accepting the rights and job descriptions of the interns
		Supportive atmosphere
	Revision of the implementation of the educational curriculum	Standard internship schedule
		Supervision and evaluation approaches
		Focus on clinical mastery process
	Retraining courses for challenging skills	ECG interpretation and cardiac care
		Pharmacological issues
		Medical orders and prescriptions
	Creating learning opportunities	Daily case discussions
		Self-study
		Learn from peers
		Learning on social media
		Reflection sessions on clinical errors
	Professionalization	Improvement in communication skills
		Improving team work
		Involving nursing interns in the care process
		Conscience and morality
		Empowering role models
	Facilities and requirements	Welfare amenities for interns
		Access to the internet and textbooks

### Theme: strategies to prevent medical errors during internship

This theme reflects the different approaches that should be applied to prevent medical errors by nursing interns. Six categories were extracted from the main theme of “strategies to prevent medical errors during internship”. These included “Professional acceptance and support”, “Revision of the implementation of the educational curriculum”, “Retraining courses for challenging skills”, “Creating learning opportunities”, “Professionalization”, and “Facilities and requirements”.

### Category 1: Professional acceptance and support

In this category, nursing interns’ experience shows that the environment in which they work contributes to medical errors. Hostile environment, lack of respect for students’ rights, and heavy workloads placed on them are causes of medical errors. This category includes two subcategories:

#### Accepting the rights and job descriptions of the interns

Many interns believed that the root cause of clinical errors was lack of respect for students’ rights and the imposition of heavy workloads that do not fit with their job description. Medical errors occur when interns are given many responsibilities, even being assigned non-nursing tasks or administering medications to all patients on certain shifts. Participant 8 explained:

*“I was shocked that a nurse with two or three patients expected me, a student who was not familiar with all the medications, to administer all the medicines to the patients. Many times we were too tired to give the medicines patients”*.

Other interns said:

*“It’s very disappointing to see a nurse who was a student until last year abusing interns. In the ward, I was treated like a footboy because I only did routine work and when I finally graduated, the same nurses teased me. What did you do during your internship?” Haven’t you learned this yet!?(Participants 3 and 6)”.*

#### Supportive atmosphere

According to some students, acceptance of students into the nursing team is still not popular among nurses, leading to a closed circles without the participation of nursing interns. Some students become isolated when they need help with patient care and feel dismayed by the lack of help and support. Participant 7 said:

*“Nurses don’t help us, saying, you have to be independent or we don’t have time. That feeling of being alone and without support was really painful and worrying.”*

### Category 2: revision of the implementation of the educational curriculum

Issues related to theoretical and clinical training as well as monitoring of students’ performance that may hinder students transferring from novice to expert status. This category includes three subcategories.

#### Standard internship schedule

The lesson plan and the clinical goals of the internship must be explained to the students. Additionally, considerations should be given to arranging standard work schedules and shifts based on student conditions with supervisors and clinical supervisors included in the schedule. Unprincipled arrangements for night shifts can lead to medical errors in interns. Changes in students’ sleep schedules and adjustments to accommodate these changes, as well as the inability to adhere to multiple night shift schedules, may contribute to the occurrence of medical errors. Participant 9 explained as follows:

“*Due to the COVID-19 pandemic, the internship programs were interrupted and many night shifts left nursing interns exhausted*. *We worked the night shift every other night and suffered from severe insomnia. My concentration was interrupted and my body had no more energy”.*

#### Supervision and evaluation approaches

An important issue that requires a basic change in practice is the evaluation of nursing intern’s knowledge, performance, and interactions as well as ethical considerations. The lack of adequate supervision of nursing students has made the hospital environment a place of student abuse, and some students have taken advantage of this lack of adequate supervision to neglect their duties. Participant 4 in Internal Medicine Department said:

The nurses betrayed the trust and respect of the nursing interns’ supervisors and threatened to report us to the supervisors and humiliate us if we did not complete all assigned tasks.

“A nursing intern in the intensive care unit said:

*“Students sleep in the room because they know when the supervisor will arrive, but the supervisor does not know which students are active in the room and which students are not (Participant 1) “.*

#### Focus on clinical mastery process

For some students, the three years of pre-internship training requires major changes. The inability of some students to achieve their educational goals, disparities in academic attainment, and lack of professionalism are examples that increase the risk of medical errors among interns. Participant 4 stated:

*“Scientific weakness and inadequate knowledge of student are the causes of medical errors”.*

Another intern said:

*“The way professors make students become experts in just one skill is completely wrong and erodes the confidence of other students. Do you understand what I mean? If a student successfully inserted the IV line, the instructor asked this student to insert the IV on the next attempt, too (Participant 1)”.*

### Category 3: retraining courses for challenging skills

Before starting their internship, interns should be exposed to different areas of care and nursing principles should be reviewed for them. These areas are more complex and require more practice than other areas such as electrocardiography (ECG), cardiac care, pharmacological issues.

#### ECG interpretation and cardiac care

For nursing interns, interpreting electrocardiograms, accurately diagnosing arrhythmias, and providing appropriate care has always been challenging, stressful, and confusing. Participant 3 said:

The patient went into cardiac arrest, and the interns supposed that the heart rhythm was due to the electrode leaving the patient’s chest and the monitor not working properly.

#### Pharmacological issues

Important points should be discussed before starting the internship. This is due to the similarity in the names and appearances of many different drugs, unfamiliarity with certain drugs, and the risk of life-threatening complications and medication errors in some drug categories. Pharmacology training workshops, continuing education in pharmacology, group discussions with supervisors, and review of physician orders are essential activities for interns and should be planned before starting the internship. Calculating medications, adjusting drops, and working with infusion pumps can be challenging for nursing interns. In addition, inadequate knowledge and improper routines of nursing staff as mentors can lead to pharmacological errors in interns. Participant 8 said:

*“The nurses in the ward all have bad routines. For example, they give medication syringes to the students and ask them to inject medications without allowing the students to check the doctor’s prescription to ensure that the medication, dosage, and route of administration are correct”.*

A nursing intern in the emergency department said:

*“Neither the nurse nor I knew how to calculate the appropriate dose of charcoal for a poisoned patient. We were really embarrassed (Participant 2)”*.

### Medical orders and prescriptions

The problem for interns is that they do not know how to read the doctor’s handwriting or doctor’s orders. This can lead to medication errors and treatment interruptions. Participant 7 in the pediatric ward said:

*“When I admitted a new patient, I found it difficult to read the doctor’s orders. Without the nurse’s help, I would have read the medicine incorrectly”.*

Another student in surgical ward said:

*“When I first saw the doctor’s order, I thought it was just rough lines until a nurse told me that the doctor had told to change the bandages twice a day (Participant 4)”.*

### Category 4: creating learning opportunities

The fourth category describes the strategies to reduce errors and improve student performance. Creating opportunities to learn in clinical practice and reflect on medical errors helps students to better understand dangerous situations and prevent similar incidence. This category includes 5 subcategories:

#### Daily case discussion

Daily review of more complex or less experienced cases is one way to reinforce learning. Putting what students have learnt into practice is a strategy to improve learning and reduce medical errors. To this end, mental reserve is performed by reviewing the patient’s medical records and conducting group discussions with the intern nurses and supervisors regarding prescribed medications and other crucial nursing interventions. As another approach, virtual spaces can be used to share specific cases and discuss them in groups. Participant 9 in triage department said:

*“It is imperative that interns raise these issues with their supervisors. Daily case review is good practice in the emergency room”.*

Another student said:

*“One way to improve students’ knowledge and performance is to organize group discussions. We can also use the virtual spaces to share important stories from our daily lives (Participant 1)”.*

### Self-study

Avoiding medical errors in the clinical settings not only depends on the help of the environment, managers and other nurses or interns, but the learners themselves must also try to avoid errors and improve their skills and clinical effectiveness. Self-study is an essential element of the internship process. Curiosity, inquisitiveness and a willingness to learn are qualities that nursing students must possess during their internship. Participant 10 from the Obstetrics Department said:

*“Asking questions is one way to avoid making mistakes and putting the patients at risk. On the other hand, owning a science notebook and studying it will increase knowledge and learning. I always carry it with me when I go to the ward. I write nursing and pharmacology notes “.*

#### Learn from peers

All staff involved in direct and indirect patient care can improve interns’ clinical performance and prevent clinical errors. Nurses play an important role in students’ clinical learning. Answering interns’ questions, monitoring procedures, and helping students solve problems are among the responsibilities of nurses, supervisors, and physicians at the clinic.

*“It is best to divide students into groups under the supervision of a qualified nurse. Experts guide the nursing interns. If I don’t have any knowledge about a medicine in the hospital ward, I ask the doctor, and he/she explains it to me (Participant 5)”.*

#### Learning on social media

Student learning should be associated with new technology. Interns’ knowledge will increase thanks to learning through social media and other learning approaches. Social media makes it easy for different groups of interns and different shifts to share their diverse experiences. Participant 7 said:

*“It is great to have a Whatsapp group where instructors can send ECGs and lab tests to explain and share some cases in the group. Sometimes when we encounter difficulties, we ask questions from each other and receive answers from friends”.*

#### Reflection sessions on clinical errors

Covering up a medical error without conducting a root cause analysis will result in similar incidents recurring. Several factors cause nursing interns to hide their errors. These factors include: fear of punishment or poor grades, nurses not trusting students, and being humiliated when students make mistakes. It may be useful to analyze clinical errors and error records in student groups and challenge them to prevent errors. These factors hide errors and make them impossible to track and analyze. Participant 2 said:

*“If students can report all medical errors anonymously, and if instructors and supervisors can hold meetings to analyze the causes, the number of errors will definitely decrease (Participant 2)”.*

### Professionalization

Acquiring skills needed in the clinical environment, such as communication skills, participation in teamwork, and care processes, will help enhance the professionalism of the internship nursing students and reduce the risk of errors.

#### Improvement of communication skills

If nursing interns can communicate with other staff members in the clinical setting, we can expect to see a reduction in clinical errors. Improving students’ communication skills and ability to discuss effectively with staff is one way to enhance learning and prevent medical errors. Participant 6 said:

*“Depending on each clinical department, there are places where you can talk to the nurses. If there is anything you don’t understand, they will explain it to you wholeheartedly, so you can be confident in your performance”*.

Another intern said:

*“Good communication skills prevent errors, so it is necessary to organize workshops to improve students’ communication skills before starting their internship (Participant 2)”.*

#### Improving team work

Effectively promoting teamwork among nursing interns improves the quality of internship course and prevents errors. Key measures include awarding credit for teamwork, encouraging senior students to take responsibility for the clinical performance of underperforming students, and encouraging teamwork and group participation among students of different proficiency levels. Participant 5 said:

*“I wanted to work together with students in the department to help me learn to calculate medications and interpret electrocardiograms, and even treat complicated medical cases. This allows for a better sharing of knowledge and skills between us. Nursing interns should also consider the results of teamwork to develop a sense of responsibility towards their peers”.*

#### Involving nursing interns in the care process

Accepting interns as potential and future colleagues and nurses, and delegating responsibility for patient care from admission to discharge can be a great way to reduce medical errors. This not only limits errors but also strengthens students’ sense of responsibility and knowledge. Participant 5 said:

“*Some nurses tell you to take blood from one patient and take vital signs from another. By taking care of one or two patients, it allows us to better understand the relationship between these procedures and the patient’s problems”.*

#### Conscience and morality

Nursing interns’ understanding of ethics and conscience are considered factors that help them not to make mistakes or cover up medical errors. Developing a moral sensitivity to the consequences of the errors and reporting them will lead to good decisions and reactions when errors are made during the internship. Participant 7 stated:

*“Nursing interns must have a deep understanding of nursing ethics and conscience. Issues of conscience are especially evident when administering medications to patients and even when treating infected wounds and colostomy procedures”.*

#### Empowering role models

Some nurses perform poorly as clinical mentors and role models, and learning wrong routines during an internship can have a negative impact. Inadequate knowledge and incorrect procedures lead to reduced competence of nursing staff. Participant 10 said:

*“A nurse in the emergency room did not have a safety box while trying to insert an intra-venous line. He stuck a bloody needle into the patient’s mattress”.*

Another interns said:

*“The nurse and I wanted to lavage the patient, but the nurse didn’t know how much serum to put in the patient’s stomach (Participant 1)”.*

### Category 6: facilities and requirements

To achieve the desired results during the internship, the structure must be changed to meet the basic needs of the internship. This category contains two subcategories:

#### Welfare facilities for interns

Welfare amenities and working conditions may be more likely to cause medical errors. Fatigue, hunger, lack of sleep, lack of welfare, and lack of student insurance all increase the risk of failure. Students face challenges during every shift, especially during night shifts, and negative effects of limited internship facilities include: lack of concentration, hypoglycemia, fear of medical errors, lack of involvement in procedures and fear of losing personal belongings due to lack of personal locker Participant 2 said:

*“There is no place to rest during the night shift. I woke up from a nap and gave the patients medicine. No wonder I make mistakes”.*

Another intern said:

*“We have no place to rest, no place to sit, no cupboards to put our groceries in. During the night shift, we get very hungry and our blood sugar levels drop.” In this case, I can’t concentrate at all (Participant 7)”.*

#### Access to the internet and textbooks

Internet access and textbooks are essential for students who have limited clinical experience and are likely to be exposed to previously unfamiliar drugs and procedures. Participant 1 stated:

*“The Internet is an alternative way to find answers to questions, avoid errors, get information, and communicate with other interns. In addition, Internet access and textbooks should be available in the ward and on the textbook stores”.*

## Discussion

This qualitative study suggests that the pre-internship and internship periods as well as the clinical environment create conditions that make nursing students susceptible to medical errors. The clinical context and working environment is challenging and makes interns susceptible to errors. However, the training of nursing students has a clear connection to the real work environment [38]. Six strategies were introduced in the present study. The first one is professional acceptance and support of nursing interns in the clinical setting and treating students as colleagues and a member of patient care, without violating rights in the work environment. Being accepted into a professional program is the first step toward achieving your career goals. By instilling an attitude of acceptance, respect, and trust, clinical instructors can have a significant impact on student learning and experiences, including those regarding medical errors [39]. Additionally, in the present study, the nursing interns also reported that their rights were not respected and that they did not receive any support in the clinical settings. Unreasonable expectations of students are the cause of medical errors [40]. In qualitative studies conducted in Canada and Addis Ababa, the nursing students perceived the clinical environment as a negative and unsupportive workplace [41, 42]. Lack of cooperation and incompatibility of clinical staff [42], violence [43] and neglect, discrimination [44], and violation of rights in the workplace have created an unfair clinical context [38]. In the present study, respecting students’ rights and supporting them, serves as an obstacle to oppression and abuse against them, and prevents the risk of clinical errors. Students must benefit from a supportive education system. For these nursing students to become successful nurses, it is essential to receive clinical training in a supportive environment. In addition, support from clinical staff has been mentioned as an important factor influencing students learning [42, 44]. Clinical supervision is also a means of supporting students in complex clinical environments [45], which was also reported in the present study as a strategy to reduce medical errors. The present study suggests that poor supervision and evaluation practice contribute to medical errors in nursing interns. In a study by Bam et al., 77% of students cited inadequate supervision in the clinical setting as a cause of medical errors [9]. Clinical training is an important part of nursing education and students can use theoretical knowledge at internship centers; therefore, supervision of this training course is mandatory. Supervision of clinical training can be associated with improved clinical skills and success of clinical education, student professionalization, personal and professional development and globalization of the nursing field. It constitutes a means of demonstrating clinical competency and a bridge between theory and clinical practice. The most important factor in the success of clinical centers is quality supervision of nursing students [45]. Students’ behavior and performance in the environment undergo changes that can have a negative impact on their learning and professional performance [46]. This is why it is important to closely monitor clinical education in order to guide students towards clinical mastery.

The third strategy is to take retraining courses for challenging skills. In the present study, the students indicated a need to review complex skills before starting an internship as important to avoid medical errors. Prior to internship, it is necessary to complete training courses in challenging skills such as electrocardiogram interpretation and cardiac care, pharmacology, and prescription reading. In a qualitative study conducted in Iran, nursing students were unprepared to enter the clinical environment in terms of knowledge, practice, and clinical performance [47]. Students encounter difficulties in the pre-internship phase because not all of the theoretical content they have learned can be applied in practice [48]. Hakim et al. found that spending more time for reviewing medication information and calculations was effective in reducing medication errors during internships [49]. Gorgich et al. show that calculating medications and prescribing medications are two areas that can lead to medical errors [24]. A study conducted in Ghana found that less knowledge of the procedure, inadequate knowledge of the medication, and workload were also associated with medication errors [9]. Students described pharmacy training programs as vulnerable and medication management skills were said to be underdeveloped [50]. Since prescribing medications is one of the primary responsibilities of nursing, it is necessary to learn mathematical skills to master medication dosage calculations such as fractions, ratios, and unit conversions. Many students have difficulties with the basic concepts of calculations, mental estimation, and critical reasoning in the competence of drug dosage calculations skills [51]. The electrocardiogram (ECG) is considered an important assessment tool, and accurate clinical judgment is directly related to the correct interpretation of the ECG [52]. In a study by Amini et al. in Iran, ECG interpretation skills of medical staff and students are low [53]. Research shows that a structured curriculum can improve nursing students’ ECG interpretation knowledge [54]. Another issue for nursing interns involves medical orders and prescriptions. The results show that doctors’ handwriting is often difficult to read and is the cause of many medication errors, harming patients and threatening their health [55–57]. A positive correlation between dispensing errors and illegible handwriting has been reported [56]. About a quarter of the copies were illegible and about a third were incomplete [57]. It appears that students in some fields need practice and repetition to avoid clinical performance problems when taking an online course.

The fourth strategy is to create learning opportunities. The results show that medical errors can be seen as opportunities for learning and growth [58]. In a study conducted by Akbari et al. in Iran, nurses reflection by nurses led to fewer medication errors [59]. Root cause analysis is a method for understanding errors, correcting them, and preventing them from occurring in the future [60]. Creating a culture of error perception is one way to minimize errors [61]. The study by Haye et al. shows the effect of role-playing simulation and written reflection in enhancing awareness, knowledge and skills in patient safety, and safe medication administration [62]. Another way of learning during the internship in the present study was the web-based learning method. Web-based media learning using a blended approach in health professions education can improve students’ knowledge and learning outcomes and is known by users to be useful and flexible [63]. Self-learning was presented as a medical error prevention strategy in the present study. Research by Zhang et al. in China, found that self-directed learning and workshops had a positive impact on students in clinical settings, including increased evidence-based practice, knowledge and attitudes, problem-solving skills as well as independent learning skills [64]. Evidence shows that technology and available resources are strategies to prevent errors [12]. Research by Pourteimour et al. found that a smartphone-based application can be used as a learning modality to increasing the knowledge about medical error prevention [65].

The fifth strategy is professionalization, which includes communication skills, teamwork, and conscious and moral, as well as empowering of role models. Nursing students have mentioned the importance of communication skills in the clinical setting [42]. One way to prevent errors is to provide opportunities for nursing students to communicate with nurses [40]. Nursing students in Addis Ababa described communication between students, instructors, and medical staff as a challenge and barrier to learning and a major cause of medical errors [40, 42]. A qualitative study conducted in Iran found that one way to avoid errors is to provide opportunities for communicating with nurses [40]. In a descriptive study in Turkey by Ozturk et al., 76% of nursing students reported communication problems on clinical wards, with the most common problems (69%) reported among clinical nurses and students [66]. Poor interpersonal communication with clinical staff and lack of support from instructors can have a negative impact on students learning progress [42]. Patient safety and minimizing medical errors are related to two factors: good communication and teamwork [67].

According to the present study, some nurses need to improve their competency because they cannot perform at their best in certain areas. In Addis Ababa, the students believed that the nurses were unqualified role models who simply followed work routines in a clinical setting. Nurses were described as un-conductive, uncooperative, and unprofessional, and uncooperative with nursing students in the clinical learning environments [42]. In Iran, nursing students reported being role models was one of the factors leading to student errors. They believe that some nurses lack skills, nurses work differently, and student repetition leads to errors [40, 68]. According to an Iranian study, the “wrong patterning” describes negative aspects of the nurse’s role that lead to nursing student misconduct in the clinical setting [38]. Additionally, many participants in a Canadian study reported difficulty receiving support and a lack of encouragement from nurses [41]. Nurses have a great impact on students learning. Therefore, working with competent and trained nurses can reduce interns’ stress in the clinical learning environment [38].

The sixth strategy involves welfare and facilities for nursing students during internship, which can also be a way to reduce and prevent medical errors. A qualitative study conducted in Iran reported similar findings regarding inadequate facilities and social services for nursing interns. Complaints from interns include lack of chairs and closets, lack of opportunities to rest or sit at nursing station, and fear of losing bags and personal items [48]. One review article found that tiredness, and fatigue from lack of sleep, and overwork, and environmental conditions all contribute to nurse errors [60, 69].

### Strengths and limitations

This study was conducted in southeastern Iran, where health infrastructure is limited and faces many challenges. Understanding the experiences of students from different universities can help identify strategies and preventive measures in this context and provide a basis for better planning for the future. However, qualitative research and the study context often have limitations regarding the generalizability of the data.

## Conclusions

The results of this study show that preventing medical errors requires different strategies before and during nursing internship. Error prevention strategies include educational and infrastructural programs. Theoretical and practical training and evaluation of students’ performance must be carried out according to accepted scientific standards. Before starting the internship, retraining and preparatory courses must be completed in more difficult areas such as electrocardiogram interpretation, cardiac care, and pharmacology. Accepting students as members of the health care team and future nurses can lead to respecting them and protecting their rights. Also, the student’s position at the medical centers and the student’s job description must be clearly defined, while taking into account their basic rights and needs. During the internship, with clinical instructors present at the bedside, it is necessary to be allowed to learn from medical errors. In addition to the students’ efforts to become an expert in providing nursing care, analysis and reflection on errors should be part of the curriculum during the internship.

### Practical implications

1- Healthcare institutions must ensure the provision of basic social facilities and professional support to students to prevent clinical errors.

2- Planning to empower students in difficult and challenging areas of study during the pre-internship phase will help reduce medical errors in the clinical setting.

3- Multifaceted monitoring of students’ performance and their support is essential to prevent medical errors.

### Electronic supplementary material

Below is the link to the electronic supplementary material.

Supplementary Material 1

## Data Availability

Data may be available on reasonable request from the corresponding author.
