# Supplementary material for: Strategies to prevent medical errors by nursing interns: a qualitative content analysis
Source: BMC Nurs. 2024 Jan 17;23:48. doi: 10.1186/s12912-024-01726-1 (PMC10792785; doi:10.1186/s12912-024-01726-1)
Supplement: Supplementary file 1 — Supplementary Material 1 [file 12912_2024_1726_MOESM1_ESM.docx]

**Title: Strategies to prevent medical errors by nursing interns: A qualitative content**

**Interview**

| Response | Question |
| --- | --- |
| Unfortunately, yes and if a student claims that he/she did not make an error during the internship, you should doubt the truth of his/her statement. | Have you ever experienced a medical error during your internship? |
| There are many reasons for the possibility of errors during internships. Students themselves, instructors, hospital environment, staff, etc. all seem to lead students to medical errors in hospital…. . | Can you explain more? |
| Drugs always get us into trouble. Different medications, related nursing care, the calculation of the dosage and the similar form of the drugs and…are things we have to deal with in shifts. Another challenge is that we don't know how to properly move the patient and this can cause damage to both the patient and the nursing interns....  We did not pay attention to the points that put our safety in dangers, such as not carrying a safety box while IV insertion or blood sampling, even getting a needle stick. Interpreting the ECG also puts a lot of pressure on the students and we fear that if our diagnosis is wrong, the nursing care will also be wrong. Sometimes, it is impossible to read a doctor's prescription from a medical record and this can lead us to make serious errors unintentionally…. | So what are the most common errors you make during your internship? |
| In my opinion, one of the reasons leading to medication errors is that nursing interns have to give drugs to all patients in the department; For example, the male surgery department has 46 patients; and there are actually a lot of drugs; The nurses wanted the students to give the medicine to the patients. My friend and I had to give medicine in the night shift. It made us so tired and had impact on our concentration.  In this department, even the staff themselves sometime are prone to medication errors, for example in micro set containing dextrose serum, they poured phenytoin. | Can you give an example of the cause of the medical error? |
| In my opinion, if the responsibility is commensurate with the abilities of the individual, the errors will be less...  Also, when there is nowhere to rest after doing all this, of course, the possibility of making mistakes increases. How can the interns have concentrate in the emergency room when there are no chairs to sit on and they can't eat anything that won't cause hypoglycemia? I think this fatigue has a big effect on their performance...  In a department like an emergency room, nurses don't allow interns to read a doctor's prescription on file. They simply asked the nursing interns to take a series of measurements without checking the patient's order; or medication is withdrawn by the nurse into the syringe and instruct the interns to inject the patients; I think that at least if the students see the drugs and prepare them, there will be less medicine errors.  This is a good way to double-check the medication card with the patient's medical record. Especially in the evening, they apply what is on the prescription because they assume that the morning shift medical staff has checked the doctor's prescription and applied the changes in the prescription. Sometimes there are errors and no one notices them. | In your experience, what measures can reduce or prevent medical errors during internship? |
| I have a booklet, if I don't have information about the medication I will look it up online or ask the staff... | You mentioned drugs. What would you do if you didn't know a drug? |
| The way to inject is written on the vial, I read it. If complications and treatment were not in my booklet, I searched the Internet or other students from other wards... | If the nurse was inexperienced and didn't know the answer to the question, what did you do? |
| In some departments, the presence of a supervisory instructor is necessary, such as emergency and intensive care departments. Sometimes we have questions and ask the instructors of other students.  In the presence of a supervising instructor, learning and the permanence and quality of learning increase. That is to say, when we come across a new case that I have never experienced before, instructors can teach us the cause of certain interventions. Their experiences and feedback can help us learn better and improve our ability to interact in different situations.  The director of nursing should send the intern job description to the head nurse in writing so that hospital staff cannot force the interns to perform tasks that are not part of their duties. On the other hand, the student should be considered as a department nurse and given the task of the new hospitalized or discharged patients so that interns have a better understanding of the admission and discharge process.  In some departments, nursing interns are only sent to accompany the patient for CT scan, ultrasound or test results, etc. I'm not saying that interns shouldn't do these things, but they should also learn how to take care of their patients. Take care of them and gain experience...  In my opinion, there shouldn't be too much of a gap between an internship and becoming a nurse (from a person who accompanies the patient to an experienced nurse); When we were interns, the medical staff treated us like a person who accompanies patients or drug-injector robots, and while we started working in the hospital, the nurses themselves complained about how we work. From small tasks like filling out forms to entire patient care, interns need to see and do so that later they won't have to be ashamed of not being able to do it.  In addition, during each shift, a certain amount of time should be spent reading information about hospitalized patients and the Q&A session. | How do you think the quality of the internship could be improved? |
| Now I don't see anything. Thank you for interviewing me for your research. | Is there anything else you haven't said? |
|  | Thank you very much. |
